# Efficacy of an adjunctive brief psychodynamic psychotherapy to usual inpatient treatment of depression: rationale and design of a randomized controlled trial

**DOI:** 10.1186/1471-244X-12-182

**Published:** 2012-10-30

**Authors:** Gilles Ambresin, Jean-Nicolas Despland, Martin Preisig, Yves de Roten

**Affiliations:** 1 Department of Psychiatry-CHUV, Centre for Psychotherapy Research, University Institute of Psychotherapy, The University of Lausanne, Av. de Morges 10, CH-1004, Lausanne, Switzerland; 2 Department of Psychiatry-CHUV, Centre for Psychiatry Epidemiology and Psychopathology, The University of Lausanne, Lausanne, Switzerland

**Keywords:** Unipolar depression, Inpatient, Psychoanalytic psychotherapy, Randomized controlled trial, Therapeutic alliance

## Abstract

**Background:**

A few recent studies have found indications of the effectiveness of inpatient psychotherapy for depression, usually of an extended duration. However, there is a lack of controlled studies in this area and to date no study of adequate quality on brief psychodynamic psychotherapy for depression during short inpatient stay exists. The present article describes the protocol of a study that will examine the relative efficacy, the cost-effectiveness and the cost-utility of adding an Inpatient Brief Psychodynamic Psychotherapy to pharmacotherapy and treatment-as-usual for inpatients with unipolar depression.

**Methods/Design:**

The study is a one-month randomized controlled trial with a two parallel group design and a 12-month naturalistic follow-up. A sample of 130 consecutive adult inpatients with unipolar depression and Montgomery-Asberg Depression Rating Scale score over 18 will be recruited. The study is carried out in the university hospital section for mood disorders in Lausanne, Switzerland. Patients are assessed upon admission, and at 1-, 3- and 12- month follow-ups. Inpatient therapy is a manualized brief intervention, combining the virtues of inpatient setting and of time-limited dynamic therapies (focal orientation, fixed duration, resource-oriented interventions). Treatment-as-usual represents the best level of practice for a minimal treatment condition usually proposed to inpatients. Final analyses will follow an intention–to-treat strategy. Depressive symptomatology is the primary outcome and secondary outcome includes measures of psychiatric symptomatology, psychosocial role functioning, and psychodynamic-emotional functioning. The mediating role of the therapeutic alliance is also examined. Allocation to treatment groups uses a stratified block randomization method with permuted block. To guarantee allocation concealment, randomization is done by an independent researcher.

**Discussion:**

Despite the large number of studies on treatment of depression, there is a clear lack of controlled research in inpatient psychotherapy during the acute phase of a major depressive episode. Research on brief therapy is important to take into account current short lengths of stay in psychiatry. The current study has the potential to scientifically inform appropriate inpatient treatment. This study is the first to address the issue of the economic evaluation of inpatient psychotherapy.

**Trial registration:**

Australian New Zealand Clinical Trial Registry (ACTRN12612000909820)

## Background

Depression is the largest contributor to the burden of disease in high-income countries, with further increase expected [1]. Depression may not respond to outpatient treatment and may be so severe that hospital stay may be needed [2,3]. Patients with severe symptoms benefit more from acute inpatient treatment than from day-hospital care [4]. Symptom improvement during first inpatient treatment is a significant predictor of the cumulative length of inpatient stay and the number of inpatient episodes over five years [5]. Those results are consistent with more recent literature that recommends an intensive inpatient acute treatment of depression [3,6].

Due to the shift of locus of mental health care in most Western countries towards outpatient care, research on role and content of acute intensive inpatient psychiatric care have received limited attention [7]. The vast majority of depressed inpatients receive pharmacotherapy, but receive psychotherapy less frequently [8-10]. Several meta-analyses support the advantage of combining pharmacotherapy and psychotherapy to treat outpatients with severe or complex depressive disorders [11-15]. The only review of combined therapy for depressed inpatients concluded that combined treatment appeared advantageous in therapy-resistant, chronic and severe forms of depressive disorders [16]. Its generalizability is limited by relatively small sample sizes and heterogeneity in diagnosis of depression, though. The best well-controlled study available in this field of research compared interpersonal psychotherapy and pharmacotherapy vs. pharmacotherapy and clinical management for 124 inpatients with major depressive disorder. This randomized controlled trial showed that inpatient depression-specific psychotherapy augmented pharmacotherapy [6].

Meta-analysis and mega-analysis findings support the efficacy of brief psychodynamic therapy for outpatient with depression [17-19]. While yet unsufficient, research on efficacy and effectiveness of psychodynamic psychotherapy as a inpatient treatment of depression gives some indications to foster examination of its validity. In a review of 9 German studies, inpatient psychotherapy, mostly psychodynamic, demonstrated good efficacy (average effect size, d = 0.84). Depression and obsessive-compulsive disorders showed the best results, but usually for psychotherapies of longer duration [20]. One cohort study on a sample of 83 consecutive inpatients examined the outcomes of short-term psychodynamic inpatient psychotherapy. Over the course of the 4 weeks of treatment, distress returned to normal range for 64% of patients and remained stable one year later [21]. This study had two major limitations. It was a complete analysis and it did not compare the treatment to a valid comparator. Based on a randomized control trial with a two parallel group design, one study explored the efficacy of an interpersonal brief (5 weeks) and intensive (15 individual and 8 group sessions) psychotherapy program combined with pharmacotherapy compared to medication and clinical management. Response rate (70% vs 51%) and remission rate (49% vs. 34%) were higher for psychotherapeutic group at discharge; after the three-month follow-up the relapse rate (3% vs. 25%) also favored the psychotherapeutic group; finally between treatment effect sizes evolved from moderate (at discharge) to large during the follow-up period (3 and 12 months) [6]. Results for a subsample of 45 patients with chronic depression revealed also a significantly greater reduction of depressive symptoms, as well as better global functioning [6,22].

Little systematic research has been conducted into the ideal dosage of brief psychotherapy. Evidence come from the outpatient setting. Between 12 and 18 sessions of therapy are required for 50% of patients to improve, according to a clinical significance perspective [23]. The Second Sheffield Psychotherapy Project found that 16 sessions were significantly more effective than 8 sessions for patients with severe depression [24]. The nature of the change aimed at should be taken into account, however [23]. Recovery from maladaptive interpersonal patterns, for example, typically requires higher doses of psychotherapy than does recovery from symptoms of depression or broader distress [25]. Changes at four weeks of inpatient psychotherapy are equivalent to a one-year follow-up for psychological distress but not for interpersonal problems [6,26].

Inpatient psychiatric treatment has been under great economic pressure to cut costs with the result of decrease in length of stay [27,28]. The brevity of inpatient stay has lead to discard psychotherapy and hindered examination of its potential cost-effectiveness. Research in outpatient care has found that although the cost of combination therapy in the initial treatment is substantially higher, these costs are in part offset by lower subsequent treatment costs [29-31]. Improvement during the acute phase of treatment is important because it is associated with lower subsequent costs across the full range of mental health and general medical services [32]. Currently there is no study done on the economicity of inpatient psychotherapy for depression.

In summary, current state of research support the need for well-controlled trials to examine the effectiveness and cost-effectiveness of inpatient acute treatment of depression, including adjunctive brief dynamic therapy to augment pharmacological treatment.

### Objectives

The first purpose of the study is to estimate the relative efficacy of combined inpatient brief psychodynamic psychotherapy (IBPP) and pharmacotherapy compared to medication and clinical management on short- and long-term outcomes of inpatients with severe, recurrent or chronic depression (including so-called treatment-resistant) according to the DSM-IVTR. The second objective is to study the cost-effectiveness and the cost-utility of the IBPP. The third objective is to document the specific and the combined influence of the therapeutic alliance with the individual psychotherapist and with the clinical team as mediators of patient’s change.

## Methods/Design

### Study design

**Figure 1:**
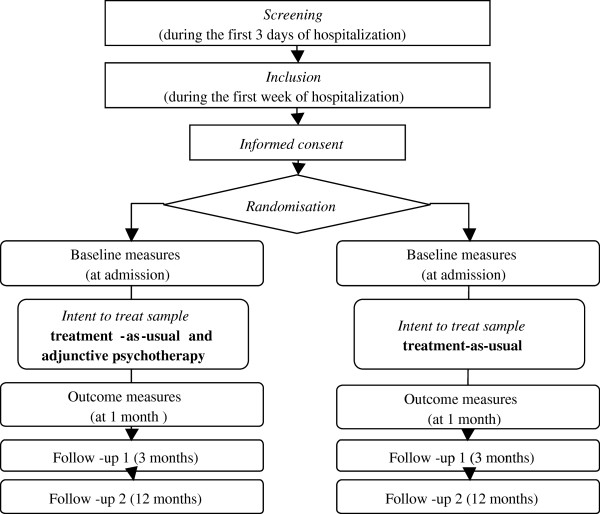
Flow chart of the research plan.

This trial is registered as: “Efficacy of an adjunctive brief psychodynamic psychotherapy compared to treatment-as-usual for psychiatric inpatients with unipolar major depressive episode” at the Australian New Zealand Clinical Trial Registy (ACTRN12612000909820). It has been peer-reviewed in the successful funding selection process by the Swiss National Science Foundation (Grant 32003B_135098/1). This protocol proposes a one month randomized controlled trial (RCT) with a 12-month naturalistic follow-up. Figure 1 illustrates the two parallel group design where patients included in the study are randomized either to (1) the intervention group (adjunctive short-term dynamic psychotherapy, IBPP), or to (2) the control treatment group, which represents the best level of practice for a minimal treatment condition usually applied to hospitalized patients (treatment-as-usual, TAU). After the one month period, patients will be referred to continuation treatment as usual.

### Ethics approval

Ethics approval was granted by the Ethics Committee of the University of Lausanne (12/04/2010).

### Inclusion and exclusion criteria

All patients admitted in the university psychiatric hospital section specialized for the treatment of mood, anxiety and personality disorders are eligible to participate in the study if they meet the following inclusion criteria: (1) Age 18–65 years old; (2) unipolar major depressive episode; (3) Montgomery-Asberg Depression Rating Scale > 18; (4) sufficient mastery of the French language.

Exclusion criteria are: (1) Any organic medical disorder, or persistent substance use/dependence which might affect brain function (memory, level of consciousness, cognitive abilities) thereby impairing the individual from participating and benefiting from psychotherapy; (2) A psychotic disorder which makes a pronounced break in reality testing chronically or intermittently likely, such as schizophrenia, delusional disorder, or bipolar manic-depression (Type I); (3) Any of the following which are considered unlikely to benefit from either treatment: axis II paranoid, schizoid or schizotypal, and borderline personality disorder, which are considered either contraindicated for some treatments, or unlikely to respond [33]; antisocial personality characterized by frequent lying, lack of guilt or remorse or history of volatile aggressiveness or violence; recent suicide attempts necessitating residential or day treatment and acute risk for suicide; other principal axis I disorder; severe cognitive impairment; previous absence of response to the study treatments.

### Power calculation

Based on previous studies, at least moderate effect sizes of psychodynamic psychotherapy on patient outcome (e.g. f = .25 for severity of depressive symptomatology) could be expected. Power analysis (with two-tailed alpha set at .05 and a power of .80) for repeated measure ANOVA indicates that 108 patients (54 in each group) are sufficient to detect the expected effects [34]. With 22% of non-completers, an *intent-to-treat sample* of 138 patients (69 in each group) is required in order to have a *completer sample* of 108 patients. All analysis will be done on the two samples.

### Intervention and comparator

#### Psychosocial Treatment-As-Usual (TAU)

A manual (Preisig M, Lustenberger Y, Fassassi Gallo S, Ambresin G, Viani I, Saraga M, Quément B: Manuel du traitement psychiatrique intégré (Treatment-as-usual) du patient déprimé hospitalisé, Unpublished manuscript, Institute for Psychotherapy, Centre Hospitalier Universitaire Vaudois and University of Lausanne; 2007) which follows the Practice guideline for the treatment of patients with Major Depression of the American Psychiatric Association [2], contains all the treatments and procedures offered to patients. Treatment includes: (1) A first interview of 45 minutes to define a treatment plan made up of patient’s, nurses’, medical, and social objectives. Therapeutic staff meets once a week to adapt it; (2) Supportive interventions of 20 to 25 minutes (clinical management), addressing psychopharmacological issues when necessary, delivered twice a week by a psychiatric resident; (3) Weekly two 30-minute encounters with nurses aiming at developing the patient’s psycho-educational skills, empowerment, and individualized treatment; (4) 6 psychoeducation group sessions; (5) Social workers, ergo-, physio-, and art-therapist interventions integrated into the treatment as required by the patient’s needs; (6) Pharmacotherapy following the rules of the World Federation of Societies of Biological Psychiatry [35].

#### Inpatient Brief Psychodynamic Psychotherapy (IBPP)

Our manualized intervention model proposes a brief psychodynamic intervention program in 12 sessions over 4 weeks (Ambresin G, de Coulon N, Despland JN: Traitement psychodynamique bref de la dépression pour patient hospitalisé, Unpublished manuscript, Institute for Psychotherapy, Centre Hospitalier Universitaire Vaudois and University of Lausanne; 2008). Sessions are 45 minutes long. It is based on the Bush, Rudden & Shapiro (2004) manual of psycho-dynamic treatment of depression to help the therapy focalize on relevant depression foci [36]. For transfe-rence, personality organization and conflictual themes the intervention is based on Despland, Michel, & de Roten (2010) manual on brief psychodynamic psychotherapy. Both manuals were adapted to the brief inpatient setting [37]. Put briefly, the 12-session psychotherapy approaches the crisis in the patient’s intrapsychic and interpersonal equilibrium that led to a hospital admission, within the context of depression [38]. It focuses on both the patient’s conscious and unconscious motives for admission. The initial hypothesis is based on the dynamic relationship established between the therapist and the patient during the first three sessions (pre-transference), on the patient’s present crisis, and on the dynamics that form the core of his/her depressive episode. Following sessions focalize further on helping the patient to gain a better understanding of the psychological factors that led to the emergence of depressive symptoms and to address his/her vulnerability to those dynamics. Final sessions address the patient’s feelings and fantasies about termination as well as the decision regarding a therapy of longer duration or long-term psychiatric treatment if necessary.

### Outcome measures

As recommended in meta-analytic reviews, various broad areas of patient’s functioning are assessed including (1) psychopathology, (2) personality, social role and quality of life, and (3) dynamic functioning which may underlie impaired functioning and contribute to vulnerability to psychiatric disorders [33]. Validated French version is available for each of the following questionnaires. Questionnaires are filled in with the help of a research assistant. Due to their depressive symptomatology, patients may face some difficulties with self-report questionnaires. Therefore we have restricted the total number of items included in the assessment battery.

#### Psychopathology

##### Depression

Depressive symptoms are the primary outcome. This is assessed by two instruments: (1) The Montgomery-Asberg Depression Scale (MADRS; [39]), a clinician rating measure in 10 items, which serves as our primary outcome measure, and (2) the self-rated version of the Inventory of Depressive Symptom (QIDS-SR_16_; [40]), a 16-item self-report measure of depressive symptoms. To allow for Kaplan-Meyer estimates of speed of response, MADRS is done on a weekly basis. The Depressive Experience Questionnaire (DEQ; Blatt SJ, D’Afflitti J, Quinlan DM: Depressive Experiences Questionnaire, Unpublished research manual, Yale University; 1979) assesses a wide range of life experiences often reported by depressed individuals but not considered symptoms of depression. Its well-established factor structure measures two primary dimensions: interpersonal relatedness and self definition.

##### Distress

The Brief Symptom Inventory (BSI; [41]) derived from the Symptom Check-List (SCL-90-R) is a widely used self-report measure of distress and psychiatric symptoms; the Global Assessment of Functioning (GAF) is a numeric scale (0 through 100) used by an independent coder to rate the social, occupational and psychological level of functioning.

##### Diagnosis

The Diagnostic Interview for Genetic Studies (DIGS; [42]) is used to collect diagnostic information. This instrument enables the collection of extensive information on the course of psychiatric conditions including mood and anxiety disorders. An updated version of the DIGS includes DSM-IV criteria [43]. The reliability of the French version was established in Lausanne for major mood and psychotic disorders [44].

##### Early trauma

Early childhood trauma is recognized as the best predictor of response to psychotherapy for severely depressed patients [45]. The childhood Trauma Questionnaire Short-Form (CTQ-SF; [46]) provides a quick, multidimensional, retrospective measure of childhood trauma with sound reliability and validity characteristics.

##### Emotions

Measured prior to therapy, alexithymia was shown to be the best predictor of residual symptoms in depressed patients who respond to short-term psychotherapy [47]. Alexithymia and other problematic patterns of emotion processing is assessed by the Dimensions of Emotional Openness (DOE; [48]).

#### Psychosocial role functioning

##### Interpersonal problems

The Inventory of Interpersonal Problems (IIP; [49]) is a self-report measure of problems in interpersonal relationships, which is widely used in psychotherapy research. We use the short version in 12 items and the main outcome is the total item-mean [50].

##### Social adjustment

The Social Adjustment Scale Self-Report (SAS-SR; [51]) assesses six areas of functioning including work, social and leisure, extended family, intimate relations (e.g., spouse), parents, and family unit, each on a six-point scale.

#### Dynamic personality functioning

##### Psychodynamic diagnosis and change

The Operationalized Psychodynamic Diagnosis is a form of multiaxial diagnostic system based on five axes: I = experience of illness and prerequisites for treatment, II = interpersonal relations, III = conflict, IV = structure and V = mental and psychosomatic disorders [52]. To measure change in this domain, we use the Heidelberg Structural Change Scale (HSCS). Studies on content, criterion, and construct validity indicate good validity for the individual axes [53].

##### Defensive functioning

The Defense Mechanism Rating Scales (DMRS; [54]) is an observer-rated method for quantitative ratings of 28 defense mechanisms. The Overall Defensive Functioning (ODF) score then summarizes the adaptive level of defensive functioning, which is capable of detecting change over time, and predicts improvement in major depressive episodes [55].

##### Therapeutic alliance

Inpatient setting requires differentiating between two different forms of alliance. Alliance with the individual therapist is assessed by the short form of the Working Alliance Inventory Short-Form (WAI-SF) in a revised version [56]. Alliance with the treatment team is assessed by the Inpatient Treatment Alliance Scale (I-TAS; [57]). For both instruments, we use the patient self-rated version.

#### Cost-effectiveness data

Collection and management of the economic data are fully integrated into the clinical data. Data will be assessed at baseline (by ad hoc retrospective questionnaire), discharge (1 month), and at follow-up after 3 months and 12 months. Measures include:

##### Costs

Information will be collected on inpatient and outpatient direct costs (hospitalizations, emergency department visits, outpatient psychiatrist, psychotherapist, physician, and other health care providers, and psychotropic and nonpsychotropic prescriptions) and indirect costs (productivity loss).

#### Quality of life (cost-utility analysis)

European Quality of Life-5 dimensions (EQ-5D) is a short standardized patient-rated instrument measuring health-related quality of life. The EQ-5D provides two important aspects: a descriptive profile based on five dimensions including mobility, self-care, usual activities, pain/discomfort, anxiety/depression, and a valuation of the profile by a visual analogue scale (EQ VAS).

#### Follow-up data (after 3 and 12 months)

Following termination of the active phase of treatment (after the 4 weeks of psychotherapy or at discharge from hospital), the vast majority of the patients are referred to an outpatient unit for additional treatment or referred to appropriate clinical services. In addition to the questionnaires, data is gathered concerning symptomatology, the type and dose/frequency of pharmacological and psychosocial ambulatory treatments, re-hospitalizations using the Longitudinal Interval Follow-up Examination (LIFE; [58]).

### Treatment integrity

#### Recording and transcription

All individual therapy sessions are audiotaped. Selected sessions (1 early session – between Ss 2 to 4 and 1 late session – between Ss 8 to 11) will be transcribed using a standardized transcription method [59]. Adherence and competence to IBPP will be assessed on selected sessions with the following instruments: (1) The *Psychotherapy Process Q-Set* (PQS ; Jones EE: Manual for the Psychotherapy Process Q-set. Unpublished manuscript, Berkeley, CA: University of California; 1975; [60]), a rating scale assessing characteristic elements of a therapeutic session. which can be compared with the ideal prototype of specific therapeutic methods (i.e., psychoanalytic, cognitive-behavioral, family-systemic, brief psychoanalytic) An expert PQS prototype specific to the IBPP will be developed and compared to the interpersonal and CBT prototypes [61]; (2) The *Psychodynamic Intervention Rating Scale* (PIRS; Cooper S, Bond M: Manual for the Psychodynamic Intervention Rating Scale, Unpublished manuscript. Montreal: Institute of Community and Family Psychiatry, Sir Mortimer B. Davis - Jewish General Hospital; 2006), an instrument elaborated specifically to investigate psychoanalytic psychotherapies. It allows to distinguish interpretative interventions from other types of intervention, and to assess the depth of understanding of interpretations on a five-point scale.

#### Therapists’ selection and training

All psychotherapists (N between 10 and 15) are resident psychiatrists in an advanced or completed stage of a 4-year psychotherapy training program in psychodynamic psychotherapy and having attended training in brief psychodynamic psychotherapy and IBPP. The IBPP training course lasts two years (two hours weekly). The first year includes theoretical presentation, role playing and case presentation. The second year is devoted to group supervision. Training is delivered by three expert trainers of the Lausanne University Medical School, who are agreed supervisors for the psychodynamic psychotherapy and authors of the IBPP manual.

#### Supervision

For the IBPP group, each therapist benefits from one individual supervision each week, delivered by the same three expert therapists that give the training. For the TAU group, each psychiatrist will benefit from one hour of individual supervision each week carried out by an experienced psychiatrist of the unit.

### Procedure

#### Data collection site

The hospital section specialized for the treatment of mood, anxiety and personality disorders is a 40-bed unit, which is part of the Department of Psychiatry of the University of Lausanne. Patients are treated in a time-limited program (mean duration stay = 26.2 days; median = 20 days). This period is considered sufficient to work through the crisis situation and its determinants and to provide for adequate follow-up treatment referral when necessary. The length of the patient stay is determined by the clinical staff, independently of the research, i.e. the time of discharge does not depend on the completion of psychotherapy, or after one month for the comparison group. Only the post-treatment measurement will be after one month, which is anyway close to the mean patient stay (26.2 days).

#### Patients recruitment

All patients hospitalized in the clinical unit with a diagnosis of major unipolar depression are referred to the project, interviewed for eligibility by a research assistant (RA) and then given the intake assessment battery. Patients giving informed consent (to be randomly assigned to one of the treatment settings; to participate in the diagnostic and psychometric procedure; to agree, if necessary, that psychotherapy sessions are audio recorded; and to take part in the follow-up measurements) are then randomized in one of the two treatment groups. Assessment with the same instruments are also done after one month of treatment (in a in- or partially out-patient setting), then after 3 months and 12 months follow-up. Cases will be considered as dropout if they had less than 5 sessions. Study is currently continuing follow-up. It is closed to recruitment of participants (as of June 2012).

#### Randomization

Allocation to treatment groups is done using a stratified block randomization method with permuted block (available at http://www.randomization.com). Three stratification variables are used to ensure treatment balance [62]: age (two levels: x<=40<y), gender (two levels: M/F), and chronicity - that full criteria for a Major Depressive Episode have been continuously met for at least 2 years (two levels: Y/N).

#### Blinding

To guarantee allocation concealment, randomization was done by a totally independent researcher, and each allocation was given to the recruiting RA in a sealed envelope prior to seeing a new patient. The envelope was opened if and only if the patient is included in the protocol. Observers are blinded to treatment arm.

#### Rater training

DIGS are done by experienced raters working at the Epidemiology and Psychopathology Research Unit, directed by one co-applicant (Prof. M. Preisig). Extensive training is provided for the MADRS (one hour weekly during three months) for the RAs. At the end of the training, an inter-rater reliability of at least ICC(2,1) > .75 should be reached. Inter-rater reliability will be calculated for 20% of the cases. All raters are blind of the clinical data.

### Data analysis

Final analyses will follow an intention-to-treat strategy comparing patients in the groups to which they were originally randomized. Clinical Significance method will be used to estimate rates of response (reliable improvement), remission (clinical significance), recovery (maintained clinical significance) and relapse (deterioration) [63]. For short-term outcome, repeated-measure analyses of variance (ANOVA) and analyses of covariance (ANCOVA - controlling for pretreatment scores and patient’s age) will be used on all outcome measures. For long-term outcome, linear mixed-effects models will be preferred, as well as for therapist and alliance effects on outcome [64]. Last Observation Carried Forward (LOCF) will be used for missing data when appropriate. Latent class analysis (using Latent Gold 4.5) will be done to explore the effect of moderating variables.

Cost-effectiveness analysis will be done in collaboration with the Institute of Health Economics and Management (IHEM) of the University of Lausanne. An economist at the IHEM will be in charge of the data analysis. The economic evaluation will be conducted from the perspective of the health system. Primary outcome will be remission in depressive symptomatology at 12 months. The comprehensively measured service costs between the two treatments will be compared with the difference in change in the primary outcome measure and with the difference between the treatments in QALYs gained.

## Discussion

Despite the large number of studies on treatment of depression, there is a clear lack of controlled research in inpatient psychotherapy during the acute phase of a major depressive episode. Research on brief therapy is important to take into account current short lengths of stay in psychiatry. The current study has the potential to scientifically inform appropriate inpatient treatment. Psychodynamic psychotherapy has shown some promising signs of efficacy but more controlled studies are strongly needed in order to be empirically validated [65]. This is a very important issue, at least for European countries like Switzerland, France or Germany where psychoanalytic psychotherapy remains the most practiced form of therapy. It may also provide indications of appropriate inpatient treatment to countries where inpatient psychiatric research has become difficult due to economic pressure. As for antidepressant drugs and various types of psychotherapy, psychodynamic psychotherapy is unlikely to be a universal therapy for depression and progress depends on identifying its most appropriate ecological niche [66].

This study is the first to address the issue of the economic evaluation of inpatient psychotherapy. Psychotherapy is costly in time and money; thus evidence of the cost-effectiveness and cost-utility of a short adjunctive psychotherapy as an alternative treatment to longer hospitalization or outpatient aftercare would be an important healthcare finding.

The project has a direct impact on the functioning of the inpatient clinical unit. The implementation of a manualized practice (for psychotherapy and psychiatric treatment-as-usual) will help to improve the organization of the inpatient care and to better structure the clinicians’ training.

### Strengths and limitations

This research has notable strength in its randomized controlled design. It compares the intervention to the state-of-the-art inpatient treatment of depression [10]. Treatments are manualized and adherence to the psychotherapy is monitored. Most important potential confounders predicting depression outcome are assessed. Despite these strengths, this research has some important limitations. It doesn’t compare the intervention to another active treatment. The intervention is added to the treatment-as-usual. One might argue that patient in the intervention arm will get more therapeutic attention. However, from a quantitative point of view, 12 hours of therapy over 4 weeks of hospitalisation doesn’t add much of therapeutic attention. Furthermore, we believe it would be unethical to compare psychotherapy and clinical management to medication and clinical management. First, medication and clinical management is probably the most used treatment for inpatients with major depressive episode. Second, evidence suggest that a combined treatment is recommended for severe depression [6,18]. The 12-month follow-up is naturalistic. Most patients will benefit of ongoing pharmacological and/or psychotherapeutic treatment in both groups after hospital discharged.

### Future research

Within a total health care delivery system, it is important to optimize the integration of inpatient and outpatient services. The next step in this project will be to select patients according to their response to the inpatient treatment and control for the outpatient psychotherapeutic after-care, as this has been neglected up till now [21].

From a research perspective, if some evidence that IBPP is effective were to be found, it would then be interesting to have a better understanding of the processes by which IBPP achieves its results. The data gathered in this trial will constitute a “gold mine” for process-outcome studies in psychodynamic psychotherapy.

## Abbreviations

IBPP: Inpatient Brief Psychodynamic Psychotherapy; TAU: Treatment-as-usual.

## Competing interests

All authors declare that: (1) No author has support for the submitted work; (2) Authors have no relationships that might have an interest in the submitted work in the previous 3 years; (3) their spouses, partners, or children have no financial relationships that may be relevant to the submitted work; and (4) Authors have no non-financial interests that may be relevant to the submitted work.

## Authors’ contributions

JND had the idea of the research. YdR, JND and GA jointly formulated the research question. YdR, JND, GA and MP collaboratively developed the design and implemented the trial. JND and MP assured the strategic coordination of the project, while GA coordinated research and clinical teams. YdR wrote the research protocol and GA drafted the manuscript of this article. MP drafted the manual for pharmacotherapy. GA drafted the manual for psychotherapy. YdR defined the statistical methods. YdR supervised data collection and data entry. All authors read and approved the manuscript.

## Pre-publication history

The pre-publication history for this paper can be accessed here:

http://www.biomedcentral.com/1471-244X/12/182/prepub
